# Enhancement of the sensitivity of single band ratiometric luminescent nanothermometers based on Tb^3+^ ions through activation of the cross relaxation process

**DOI:** 10.1038/s41598-020-68145-5

**Published:** 2020-07-07

**Authors:** Joanna Drabik, Robert Kowalski, Lukasz Marciniak

**Affiliations:** Włodzimierz Trzebiatowski Institute of Low Temperature and Structure Research, Wrocław, Poland

**Keywords:** Nanoscale materials, Optical physics, Physical chemistry

## Abstract

The description of luminescent processes and their thermally induced changes, that may be also influenced by the optically active ions concentration, and thus by the various inter-ionic processes, is the key to the improved development of luminescence thermometry. A phosphor doped with only trivalent terbium ions was described, which, by using two excitation lines fitted to the ^7^F_6_ → ^5^D_3_ and ^7^F_5_ → ^5^D_3_ transitions, shows a luminescent signals with the opposite characteristics of intensity changes as a function of temperature. By modifying the concentration of Tb^3+^ ions, the probability of {^5^D_3_, ^7^F_6_} ↔ {^5^D_4_, ^7^F_0_} cross-relaxation was being altered, which turned out to have a beneficial effect on the properties of the described nanothermometers. The ratio of intensities for both excitations was found to be temperature dependent, which resulted in high relative sensitivities of temperature readout reaching 3.2%/°C for 190 °C and not reaching values below 2%/°C in the broad range of the temperature. Extensive decay time measurements for ^5^D_3_ and ^5^D_4_ emissive levels were presented and the variability of both rise- and decay times as a function of terbium concentration and temperature was investigated. Thanks to this, conclusions were drawn regarding thermally dependent optical processes occurring in a given and similar systems.

## Introduction

The technique of high-resolution noncontact temperature measurement in the single-band-ratiometric (SBR) approach has been recently developed^1–6^. It is an application of luminescence thermometry technique^7–10^ which requires only one type of optically active center in any nanocrystalline matrix, and enables temperature readout with high resolution and significant relative sensitivity (S_r_). This approach has all the advantages of a ratiometric measurement^11–22^, and at the same time does not show susceptibility to partial signal interference by the possible changes in the transmission characteristics of the medium^23^. The ratio of two spectrally separated bands is receptive to artificial alterations through various, not only thermal, factors modifying their shape, especially in biological media^24^. The phosphors used in this approach may be based on different luminescent dyes or ions, whose emission intensity depends on the temperature in opposite ways, depending on the excitation line used. There can be many reasons for different temperature dependencies. The various conformational changes occurring in organic phosphors that result in a change in the shape of the absorption bands should be mentioned. In addition, it is possible to use an energy mismatch between energetic states, so that a given excitation line does not have a regular dependence of the resultant emission intensity on the temperature. Additionally, different types of energy transfers between optically active centers are possible, which may cause different dependence of emission intensity depending on the center which is excited using a given excitation wavelength. Nevertheless, one of the most promising physical processes, which leads to the difference in the thermal dependences of the absorption bands intensities, is excited state absorption (ESA).

Green fluorescent protein showing 510 nm emission, whose luminescence upon 400 nm and 480 nm excitation wavelengths proved to be a good temperature dependent parameter, was used in HeLa cells to visualize thermogenesis^1^. Due to conformational changes occurring at a temperature of about 37 °C, a notable S_r_ (2.57%/°C) was obtained around this biologically relevant temperature. However, inorganic phosphors have also been implemented, since they do not exhibit photobleaching in most cases, and they are more resistant to chemical and physical influences. The transition metal Cr^3+^_,_ with its red emission around 720 nm was also investigated for the SBR approach^2,25^, taking advantage of the thermally induced spectral shift of its absorption band. Cr^3+^ emission intensities upon 590 nm and 610–650 nm excitation wavelengths reveal different dependence on the temperature. This approach showed the promising characteristics, despite the relatively lower S_r_ of 0.3–0.35%/°C. Further examples of SBR nanothermometers were based on lanthanide ions, taking advantage of their high emission brightness and narrow absorption and emission lines. Moreover, a well-defined energy structure of the lanthanides facilitates efficient ESA process and allows for the selection of such wavelengths that may result in quite different temperature dependence of emissions^26,27^. Among different lanthanide ions examined in SBR approach, the Eu^3+^ ion is one of the most extensively described in the literature^3–5^. Excitation wavelengths matching its ground- (GSA) and excited state absorption (ESA), causing ^7^F_0_ → ^5^D_0_ and ^7^F_2_ → ^5^D_0_ transitions, respectively, were utilized. The resulting red emissions of opposite thermal dependence, enabled noncontact temperature readout with S_r_ of 1.68–2.31%/°C. On the other hand, the relatively large energy gap between the ground and the first excited states of Tb^3+^ ions hinders the efficient ESA process. Therefore the number of ESA-based SBR luminescent thermometers in this case is strongly limited^28,29^. Our previous work^6^ showed the use of Tb^3+^ ions in the SBR approach using excitation matched to the ^7^F_6_ → ^5^D_4_ transition from the ground level and ^7^F_5_ → ^5^D_4_ transition from the first excited level, and a significant S_r_ for this approach of up to 5%/°C was reached. That choice of the excitation wavelengths was dictated by the desire to simplify the possible thermally dependent processes in the studied system. A priority was to demonstrate a compatibility of the experiment with the theory based on the system of state equations. An in-depth analysis of the thermally dependent physical processes which affect ESA, enabled the optimization of the system in order to develop highly sensitive SBR luminescent thermometer. However, a different selection of the excitation wavelengths presented in this work facilitates the concentration quenching of the luminescence related to the Tb^3+^ content-activated cross-relaxation (CR) process. This is not only a much more universal description that covers all possible luminescent processes both intra- and inter-ionic, in systems based on any lanthanide in general, but above all a way to enhance the S_r_ values in a wide temperature range. An investigation of the impact of enabling energy transfer paths between ions on the achieved S_r_ values and other thermometry-related parameters, is an issue of a great importance. In this work, we present a versatile analysis of the effect of interionic processes on the operation of SBR nanothermometers. The two-ion {^5^D_3_, ^7^F_6_} ↔ {^5^D_4_, ^7^F_0_} CR process was activated by selecting the appropriate excitation lines matched to the transitions from the ground- and excited level, ^7^F_6 _→ ^5^D_3_ and ^7^F_5 _→ ^5^D_3_, respectively. Due to extensive analysis of luminescence decay times, the relationship between the content of Tb^3+^ ions in the KLa_1−x_Tb_x_P_4_O_12_ nanocrystals and the probability of the CR process, was verified. It was shown that by activation of this process, the S_r_ of nanothermometers is enhanced. Also, thanks to the favorable Judd–Ofelt parameters for transitions matched to the chosen excitation lines, highly intense luminescent signals were obtained. In addition, in order to understand the impact of all the processes occurring in the studied system, an experimental and theoretical analysis of the change in their probabilities depending on temperature was carried out.

## Methods

KLa_1−x_Tb_x_P_4_O_12_ nanocrystals with different concentrations of Tb^3+^ ions were synthesized by the co-precipitation method. Due to similar ionic radii, Tb^3+^ ions are substituted for La^3+^. The concentrations of x = 0.02, 0.05, 0.1, 0.2, 0.5, 1 (with no La^3+^ in the crystal matrix) were selected for the study. The following precursors were used for the synthesis: ammonium phosphate dibasic ((NH_4_)_2_HPO_4_ of 99.99% purity from Sigma Aldrich), lanthanum oxide (La_2_O_3_ of 99.999% purity from Stanford Materials Corporation), terbium oxide (Tb_4_O_7_ of 99.99% purity from Stanford Materials Corporation), potassium carbonate (KCO_3_ of 99% purity from Avantor) and nitric acid (HNO_3_ of 65% purity from Avantor). In the first step, a stoichiometric amount of lanthanide oxides was prepared per 1 g of the product. Using excess nitric acid, they were converted to nitrates, followed by a three-times recrystallization process to get rid of a remained nitric acid. Later, in a separate beaker, an aqueous potassium carbonate solution was prepared, to which a drop of nitric acid was also added, and the solution was recrystallized. The contents of both beakers were combined and poured into an aqueous solution of ammonium phosphate dibasic, which resulted in the appearance of a visible precipitate. The samples were thoroughly mixed and placed in an oven at 90 °C for 2 days to dry. The last step was to anneal the grated powder in an oven at 450° for 3 h. After the synthesis was completed, the nanocrystalline powder samples were again ground in a mortar.

The FLS980 spectrometer from Edinburgh Instruments equipped with a R928P side window photomultiplier tube from a Hamamatsu detector, was used to carry out measurements of photoluminescence decay curves and excitation spectra utilizing a micro-flash lamp and a halogen lamp, respectively. The emission spectra were measured using Silver-Nova Super Range TEC Spectrometer from Stellarnet of 1 nm spectral resolution and 377 or 413 nm excitation lines from an OPOLLETE 355 LD Optical Parametric Oscillator. The thermal evolutions of both excitation and emission spectra were measured using THMS 600 heating stage from Linkam (0.1 °C temperature stability and 0.1 °C set point resolution) to control the temperature.

Luminescence decays were measured using a Libra laser from Coherent (1 mJ, 89 fs), an OPerA-Solo Optical Parametric Amplifier and a Streak Camera from Hamamatsu.

All calculations were carried out using proprietary software written in the Python programming language, using SciPy^30^ and NumPy (Copyright 2005–2020, NumPy Developers) modules.

## Results and discussion

The synthesized KLa_1−x_Tb_x_P_4_O_12_ show high compliance with the reference data for potassium lanthanum tetraphosphate^6^ (Supplementary Fig. S1a). All the investigated KLa_1−x_Tb_x_P_4_O_12_ nanocrystals crystallize in a monoclinic structure with P12_1_1 symmetry (Supplementary Fig. S1b) and no concentration-related alteration were found. Crystallographic sites of La^3+^ ions may be easily substituted by the Tb^3+^ ions due to the similarity in ionic radii, 0.130 and 0.118 nm, respectively. The large distances between these crystallographic positions of C_1_ point symmetry is the reason that even very high concentrations of Tb^3+^ ions (reaching up to 100% substitution for La^3+^ ions) can show luminescence without much effect of concentration quenching of emission. Measurements of Raman spectra confirmed a very high maximum energy of phonons of about 1,180 cm^−1^, which is beneficial in the case of SBR^6^. KLa_1−x_Tb_x_P_4_O_12_ nanocrystals showed only slight changes in the shapes of excitation and emission spectra, also the decay time measurements for excitation and emission from ^5^D_4_ level proved to be only slightly dependent on the concentration of Tb^3+^ ions^6^. In the excitation spectra, a number of *f*–*f* intraconfigurational transitions can be observed in the range of 300–500 nm (Supplementary Fig. S1c). In the range of about 377 nm a band corresponding to the ^7^F_6_ → ^5^D_3_ transition is visible, which has a relatively high intensity, whereas between 400 and 480 nm at room temperature, no additional excitation bands are observed. The emission spectrum of Tb^3+^ ions exhibiting bright green luminescence consists of a series of narrow bands at 543, 585, 620, 650, 670 and 680 nm, which are associated with ^5^D_4 _→ ^7^F_J_ transitions for J = 5, 4, 3, 2, 1 and 0, respectively. The band corresponding to the ^5^D_4 _→ ^7^F_6_ transition was not recorded around 485 nm because of the 500 nm long-pass filter used. The most intense emission band is located around 543 nm and it is associated with the ^5^D_4 _→ ^7^F_5_ transition. In the range of shorter wavelengths at lower concentrations, an emission from ^5^D_3_ level could be also observed. However, due to the increment in the probability of the {^5^D_3_, ^7^F_6_} ↔ {^5^D_4_, ^7^F_0_} CR process as the concentration of Tb^3+^ increases, these bands become less visible.

**Figure 1:**
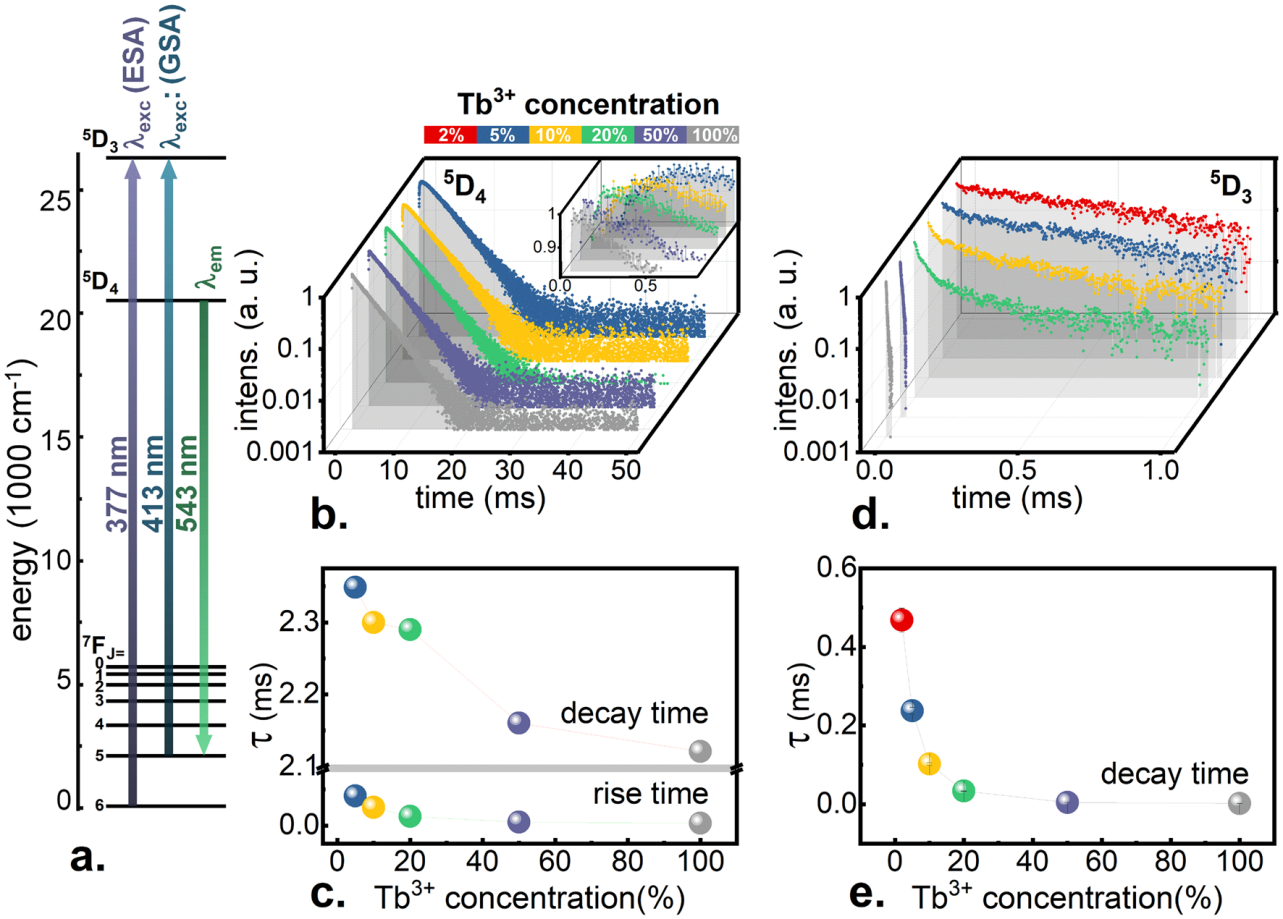
Energy level diagram with the most important transitions and the corresponding wavelengths marked—(**a**). Luminescence decay curves (λ_exc_ = 266 nm, λ_em_ = 543 nm) of KLa_1−x_Tb_x_P_4_O_12_ with different Tb^3+^ concentrations—(**b**). Rise times and decay times calculated from the decay curves (λ_exc_ = 266 nm, λ_em_ = 543 nm)—(**c**). Luminescence decay curves (λ_exc_ = 377 nm, λ_em_ = ∫(400–450 nm)) of KLa_1−x_Tb_x_P_4_O_12_ with different Tb^3+^ concentrations—(**d**). Decay times calculated from the decay curves (λ_exc_ = 377 nm, λ_em_ = ∫(400–450 nm))—(**e**).

To find out the exact effect of concentration on luminescence dynamics, measurements of decay curves were carried out. The results obtained for monitoring the λ_em_ = 543 nm wavelength corresponding to the ^5^D_4_ → ^7^F_5_ transition (see Fig. 1a) upon an excitation matched to the higher levels are shown in Fig. 1b. A slight shortening of the decay times is visible (Fig. 1c). The ^5^D_4_ level is characterized by relatively long lifetime from 2.35 ms for KLa_0.95_Tb_0.05_P_4_O_12_ to 2.12 ms for KTbP_4_O_12_. Such a small concentration effect was expected since the energy gap between the ^5^D_4_ level and the lower ^7^F_0_ level is high enough to hinder its multiphonon relaxation. Therefore, shortening the decay times of this level on the order of only 10% of its value is the result of an increase in the probability of energy loss during migration, when for example, non-luminescent defects are encountered. Due to the fact that the ^5^D_4_ level is populated from the ^5^D_3_ state, the described decay is preceded by the rise time of luminescence intensity (Fig. 1b, inset). Observed decrease of the rise time with Tb^3+^ concentration, results from the growing probability of {^5^D_3_, ^7^F_6_} ↔ {^5^D_4_, ^7^F_0_} CR process, which quenches the ^5^D_3_ state population. Due to the fact that the emission from the ^5^D_3_ level shows significantly shorter lifetimes in µs range, its luminescence decay curves were measured using a spectrally resolved streak camera (Supplementary Fig. S2). By integrating the recorded intensity in the 400–450 nm spectral range (corresponding to the ^5^D_3_ → ^7^F_J_ transitions) in time domain, luminescence curves of the ^5^D_3_ level were obtained for all KLa_1−x_Tb_x_P_4_O_12_ nanocrystals (Fig. 1d). Decay times were found to be significantly shortened with an increase in the Tb^3+^ concentration, by two orders of magnitude from 468 µs for KLa_0.98_Tb_0.02_P_4_O_12_ to 2 µs for KTbP_4_O_12_ (Fig. 1e), which is the result of the growing influence of {^5^D_3_, ^7^F_6_} ↔ {^5^D_4_, ^7^F_0_} CR process. This phenomenon is more efficient for higher concentrations of Tb^3+^ and causes an efficient nonradiative pumping of the ^5^D_4_ level at the expense of the ^5^D_3_ level. Therefore, it could be concluded that with increase in the content of Tb^3+^ in the nanocrystalline matrix, CR process becomes more prominent. Moreover, its high probability should facilitate ESA process, because CR-related population of the ^7^F_0_ state is followed by fast nonradiative transitions to the ^7^F_5_ state.

**Figure 2:**
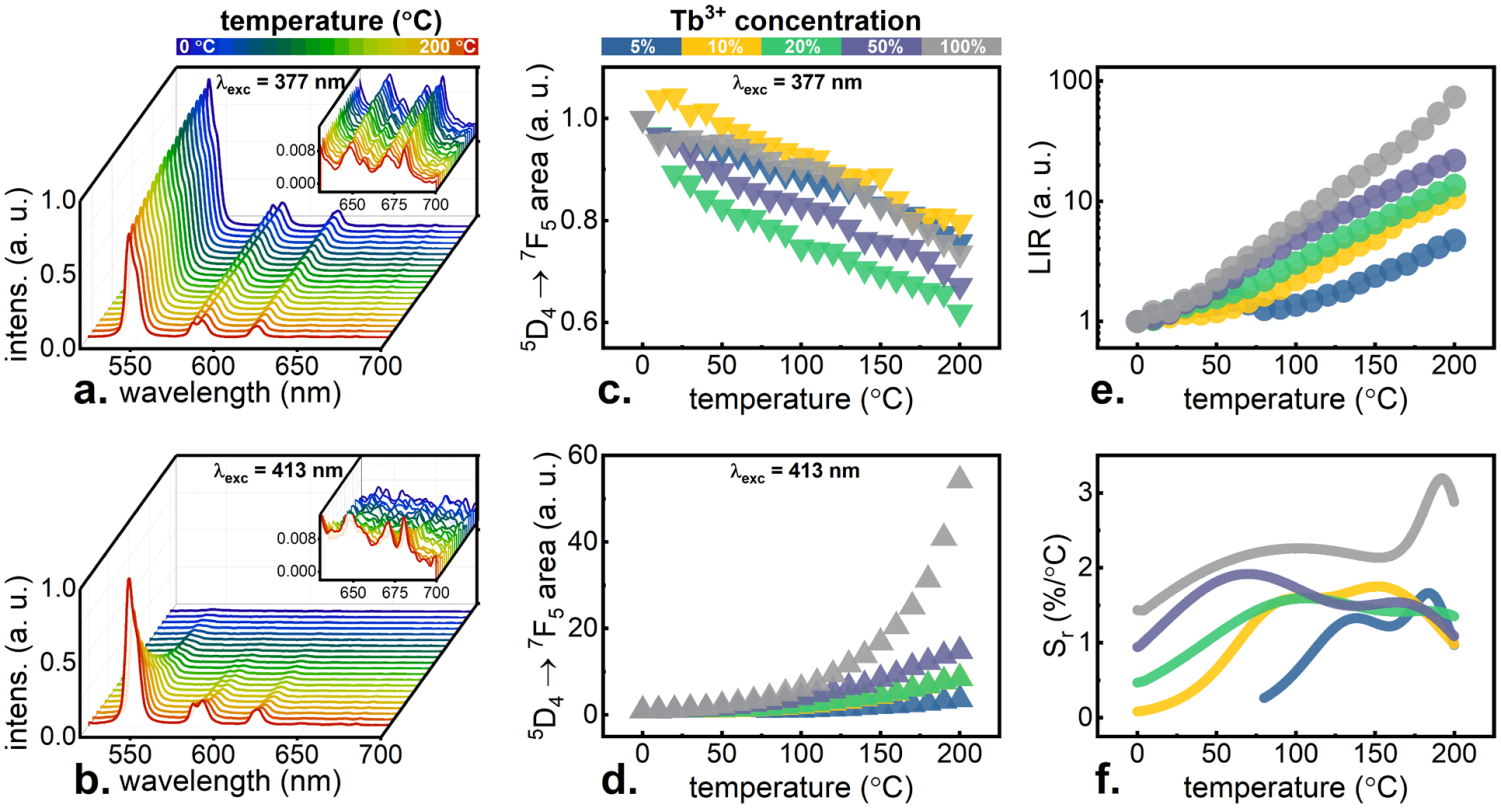
Thermal evolution of the emission spectra under excitation matching GSA—(**a**) and ESA—(**b**) for a representative KTbP_4_O_12_ nanocrystals measured using optical filter long-pass 500 nm. Area of the peak fitted to ^5^D_4 _→ ^7^F_J_ transition under excitation matching GSA—(**c**) and ESA—(**d**) for KLa_1−x_Tb_x_P_4_O_12_ with different Tb^3+^ concentrations (normalized to the first value). Single band luminescence intensity ratio—(**e**) and sensitivities calculated from curves fitted to LIR—(**f**) for KLa_1−x_Tb_x_P_4_O_12_ with different Tb^3+^ concentrations.

The influence of Tb^3+^ concentration increase, and thus the CR probability enhancement, on the thermometric properties of KLa_1−x_Tb_x_P_4_O_12_ nanocrystals was verified by characterizing their emission properties in a wide temperature range. Figure 2a,b show the results of the representative measurements recorded using 377 nm excitation line matched to ^7^F_6_ → ^5^D_3_ transition (GSA) and 413 nm adjusted to ^7^F_5_ → ^5^D_3_ transition (ESA), respectively. Upon GSA excitation, the characteristic Tb^3+^ emission bands can be observed, emission intensity of which decreases at elevated temperatures. On the contrary, in the case of ESA excitation, only a very weak emission was observed at 0 °C. However, a gradual increase of temperature caused a significant enhancement of Tb^3+^ emission intensity. For all the investigated nanocrystals, these results were analyzed as a function of changes in the surface area of the peak fitted to the band corresponding to the strongest ^5^D_4_ → ^7^F_5_ transition around 543 nm^6^. For stimulation with GSA-matched wavelength, the decrease by around 30% of the initial emission intensity was found without any significant influence of Tb^3+^ concentration as shown in Fig. 2c. For the ESA-matched excitation wavelength, a strong concentration effect is visible—for low dopant concentrations the intensity changes only several times in the range of 0–200 °C, while for KTbP_4_O_12_ the enhancement up to 54 times was observed (Fig. 2d). The opposite monotonicities of the ^5^D_4 _→ ^7^F_5_ (the terms were sequentially numbered from the ground ^7^F_6_ level and ^7^F_5_, ^7^F_4_, ^7^F_3_, ^7^F_2_, ^7^F_1_, ^7^F_0_, ^5^D_4_ levels to ^5^D_3_ level by numbers i from i = 0 to i = 8) emission band upon 413 nm and 377 nm excitation confirm that their emission intensity ratio:

1 $$LIR = \frac{{I_{7 \to 1}^{excESA} }}{{I_{7 \to 1}^{excGSA} }}$$

is a very reliable temperature dependent parameter (Fig. 2e). In the case of x = 0.05 Tb^3+^ ions LIR increases by around eight times in the temperature range under investigations, while for the stoichiometric KTbP_4_O_12_ counterpart, the enhancement by two orders of the magnitude was observed. This strong concentration effect is directly related to the increase in the probability of CR for the increase in the content of Tb^3+^ ions in the matrix. In order to perform a quantitative analysis, the relative sensitivity of Tb^3+^ based SBR luminescent nanothermometer was calculated as follows:

2 $$S_{r} = \frac{1}{LIR}\frac{\Delta LIR}{{\Delta T}}$$

In the case of S_r_, a dependence on concentration analogous to LIR is visible (Fig. 2f) and thus the highest S_r_ reaches 2–3%/°C in the whole tested temperature range for KTbP_4_O_12_. The increase of Tb^3+^ concentration affects the values of S_r_ also in the case of direct ^5^D_4_ excitation^6^. However, in the present case, the maximal values of S_r_ were obtained at relatively low temperatures, and their decrease with temperature was noted. Therefore, the thermal dependence of S_r_ for ^5^D_3_-excitation-approach enables the widening of the temperature range in which SBR temperature readout can be obtained with high S_r_ in respect to the ^5^D_4_-excitation case. This opens up a completely new application possibilities for this phosphor. Additional benefits comes from the fact that both temperature readout modes are offered by the same nanocrystalline phosphor, for example, the effect of uneven location or aggregation of two different types of optically active centers is avoided. Moreover, it is very important, that in this case, even at the lowest concentrations, where S_r_ is much lower around 1%/°C, the emission brightness in the case of ESA was much higher than in the case of ^5^D_4_ level-matching excitation wavelengths, and thus the temperature readout uncertainty decreases in such a measurement configuration.

**Figure 3:**
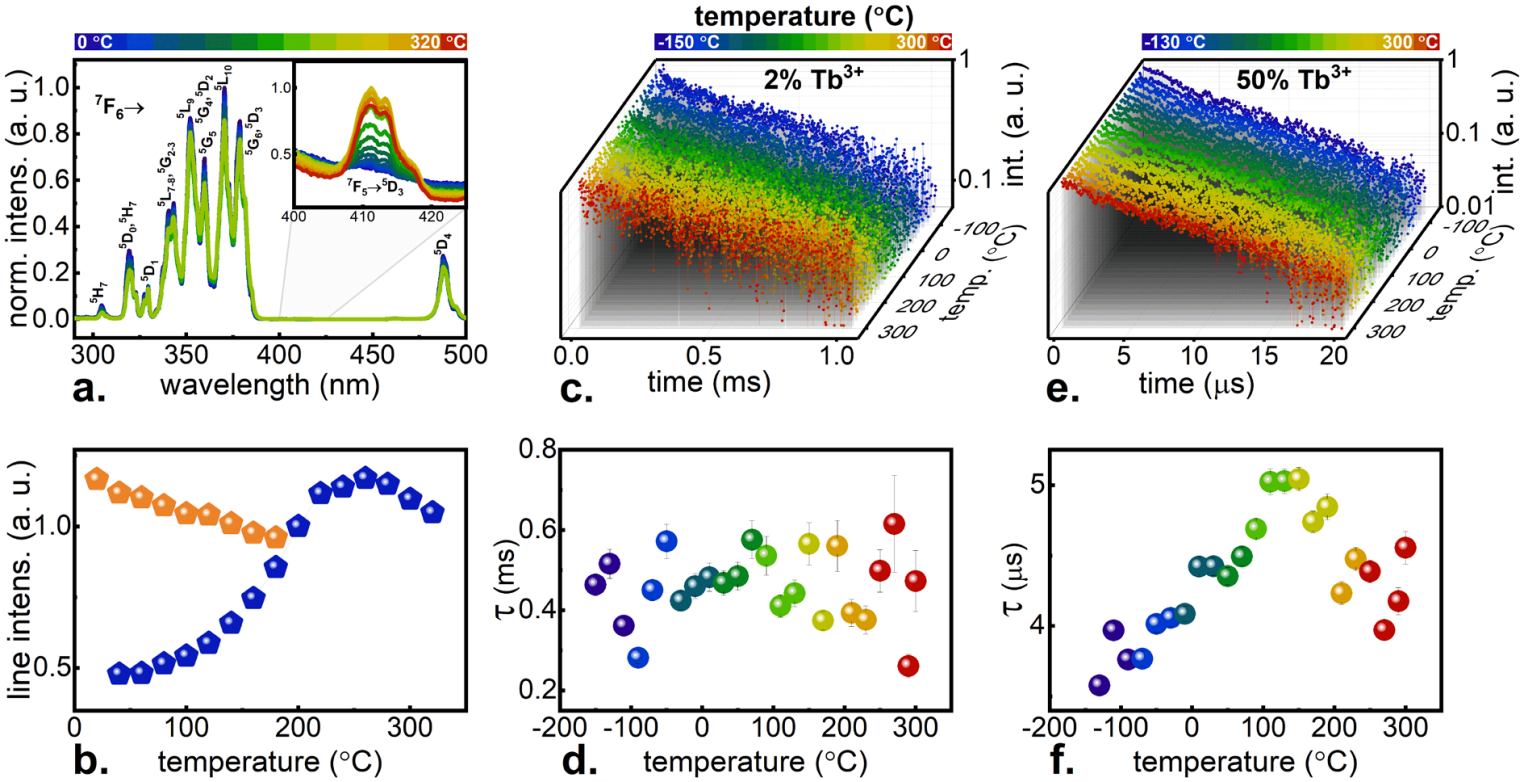
Thermal evolution of the excitation spectra (the inset shows the 400–425 nm wavelength range measured in a broader temperature range; the scale was changed approximately 450 times) measured using optical filter short-pass 500 nm—(**a**). Thermal evolutions of the chosen lines from the excitation spectra: orange symbols for 377 nm line and blue symbols for 413 nm line—(**b**). Thermal evolution of luminescence decay curves (λ_exc_ = 377 nm, λ_em_ = ∫(400–450 nm)) for KLa_0.98_Tb_0.02_P_4_O_12_—(**c**). Decay times calculated from the decay curves of KLa_0.98_Tb_0.02_P_4_O_12_ at different temperatures—(**d**). Thermal evolution of luminescence decay curves (λ_exc_ = 377 nm, λ_em_ = ∫(400–450 nm)) for KLa_0.5_Tb_0.5_P_4_O_12_—(**e**). Decay times calculated from the decay curves of KLa_0.5_Tb_0.5_P_4_O_12_ at different temperatures—(**f**).

In order to provide a deeper insight into the understanding of the ESA in the Tb^3+^ doped system, a detailed theoretical consideration concerning the influence of dopant concentration and temperature on the particular physical processes was carried out. As it can be seen in excitation spectra measured for KTbP_4_O_12_ nanocrystals in the range of 0–200 °C (Fig. 3a), all the visible *f*–*f* bands gradually reduce their intensity with the temperature elevation. The ^5^D_3_ direct excitation band located around 377 nm decrease in intensity approximately linearly (Fig. 3b, orange symbols). This is related to the increase in electron–phonon coupling with the temperature elevation that was postulated earlier^6^. Therefore, for the use of laser lines exciting the ^5^D_3_ level, it was proposed that the probability of such laser induced transitions *W*_*LD*_ should decrease in an approximately linear way:

3 $$W_{LD:i \to j} (T) \equiv W_{LD:i \to j}^{T} = coeff_{LD} \cdot [T - T_{0} ] + W_{LD:i \to j}^{{T_{0} }}$$

In the experiments for GSA-matched excitation: i = 0, j = 8 and for ESA: i = 1, j = 8. The *coeff*_*LD*_ parameter is a fixed value and provides a linear temperature dependence of *W*_*LD*_. The value of initial $W_{LD}^{{T_{0} }}$ probability is directly dependent on the laser radiation power and the absorption cross section for a given excitation line.

At higher temperatures and in the narrower spectral range, also a band around 413 nm was recorded (Fig. 3a, inset), temperature characteristics of which are quite the opposite. As the temperature increased, this band gained intensity, and the measurement was continued up to 320 °C. However, an increase in the intensity of this band was recorded only up to about 250 °C (Fig. 3b, blue symbols), and then a slight decrease in intensity was noted, which is associated with a domination of thermal quenching processes similar to that described above. Such characteristics indicate that the observed band is not associated with absorption from the ground state, but from the thermally coupled ^7^F_5_ level to the ^5^D_3_ level. It should be noted that these measurements were not carried out using laser light, but with a halogen lamp of much lower intensity. Nevertheless, it was possible to observe the thermal evolution of ^7^F_5_ → ^5^D_3_ excitation band, which indicates that the ESA process is extremely efficient for a sample at this Tb^3+^ concentration. Many other factors certainly influenced the thermal evolution of the intensity of this excitation band, which will be discussed later. Nevertheless, it was assumed that the *W*_*LD*_ probability for a 413 nm line fitted to this transition also changed accordingly to Eq. (3), because the power of the laser used was constant, and the absorption cross-section, despite a different value than for transitions matched to GSA, also depends on the electron–phonon coupling, which in turn becomes stronger with temperature for all bands. The absorption cross-section depends on the oscillator strength of the transition and on the values of the Judd–Ofelt parameters^31^. According to Judd–Ofelt theory, the probability of transition is proportional to the sum of the vector product of Ω parameters and U parameters. Analyzing squared reduced-matrix elements (Supplementary Table S1) proportional to U parameters, and taking into account that the probabilities related to transitions from level i → j are equal to these related to transitions from level j → i, it could be concluded that the transitions to level ^5^D_4_ are two orders of magnitude less probable than to ^5^D_3_ level. Hence, the emission brightness at excitations matched to transitions to the ^5^D_3_ level is higher. It is also evident that in each case transitions from ^7^F_6_ level are less probable than from ^7^F_5_ level, what facilitates the ESA process.

A photon of radiation corresponding to a wavelength of 413 nm has an energy lower by approximately 2000 cm^−1^ than required to cause a transition from the ground level to the ^5^D_3_ level. Due to the fact that the tetraphosphate matrix has phonon energy of 1,180 cm^−1^, less than two phonons are sufficient to bridge this missing energy gap. Temperature elevation increases the probability of *W*_*pa*_ according to the Miyakawa–Dexter formalism^31^:

4 $$W_{pa:i + phonon \to j} (T) = W_{pa:i + phonon \to j}^{T} = L_{1} e^{{ - L_{2} \Delta E}} \left[ {1 - e^{{\frac{{ - \omega_{\max } }}{kT}}} } \right]^{{\frac{ - \Delta E}{{\omega_{\max } }}}}$$

This phenomenon is only taken into account for ESA-matched excitation and in this case i = 0, j = 8. The *L*_*1*_ may be understood as initial *W*_*pa*_ probability, *L*_*2*_ is a parameter, *∆E* is an energy mismatch (2000 cm^−1^ in this case) and *ω*_*max*_ is a maximum phonon energy in the matrix (1,180 cm^−1^).

The streak camera was then used to measure lifetimes for selected Tb^3+^ concentrations over a wide temperature range. The course of the ^5^D_3_ luminescence decay curves for KLa_0.98_Tb_0.02_P_4_O_12_ is shown in Fig. 3c. No significant changes are observed, which indicates that the decay time for low Tb^3+^ concentration is independent on temperature and oscillates around the 468 µs (Fig. 3d). At such a low concentration of Tb^3+^, the interaction between ions can be neglected in this case, and hence, it can be assumed that the time measured experimentally is very close to the radiative one and the noted temperature stability confirms this thesis. Therefore, it was assumed, that the probability of radiation transitions from both ^5^D_4_ and ^5^D_3_ levels *W*_*r*_ can be designated as:

5 $$W_{r:i \to j} = W_{r:i}^{{T_{0} }} \cdot \beta_{i \to j} \ne f(T)$$

In case of luminescent levels ^5^D_4_ and ^5^D_3_, the following numbers are implemented: for i = 7: j = 0,…,6 and for i = 8: j = 0,…,7; the *β*_*i*→*j*_ is the branching ratio for the radiative transition from level i to level j. The probability *W*_*r*_ of such a transition is the inverse of radiative lifetime for a given emitting level.

Analogous measurements of decay times as a function of temperature were carried out for KLa_0.5_Tb_0.5_P_4_O_12_ (Fig. 3e). Assuming that in the case of such a high concentration of Tb^3+^ ions, the emission process is additionally strongly influenced by inter-ionic CR interaction, it was assumed that the relationship between the observed decay time (Fig. 3f) and the radiative time for Tb^3+^ ions luminescence in this matrix (468 µs) is as follows:

6 $$\frac{1}{{\tau_{\exp } }} = \frac{1}{{\tau_{rad} }} + W_{cr}$$

On this basis, the CR probability *W*_*cr*_ (Supplementary Fig. S3) as a temperature function for this concentration of Tb^3+^ was analyzed, and it was found that in the range of 0–200 °C *W*_*cr*_ decreases approximately linearly. For the remaining concentrations, *W*_*cr*_ was extrapolated based on the decay times measured at room temperature (Supplementary Fig. S3). It was also assumed that for low Tb^3+^ concentrations at higher temperatures, the probability of the CR process is negligible (Supplementary Fig. S3). Therefore, it can be stated that *W*_*cr*_ has a linear dependence on temperature in the investigated range:

7 $$W_{cr:i,j \leftrightarrow k,l} (T) \equiv W_{cr:i,j \leftrightarrow k,l}^{T} = coeff_{cr} \cdot [T - T_{0} ] + W_{cr:i,j \leftrightarrow k,l}^{{T_{0} }}$$

In the case of CR process among Tb^3+^ ions, the levels that take place in it are designed as: i = 0, j = 8, k = 6, l = 7. The *coeff*_*cr*_ is a negative constant value determined by the analysis presented in Supplementary Fig. S3.

**Figure 4:**
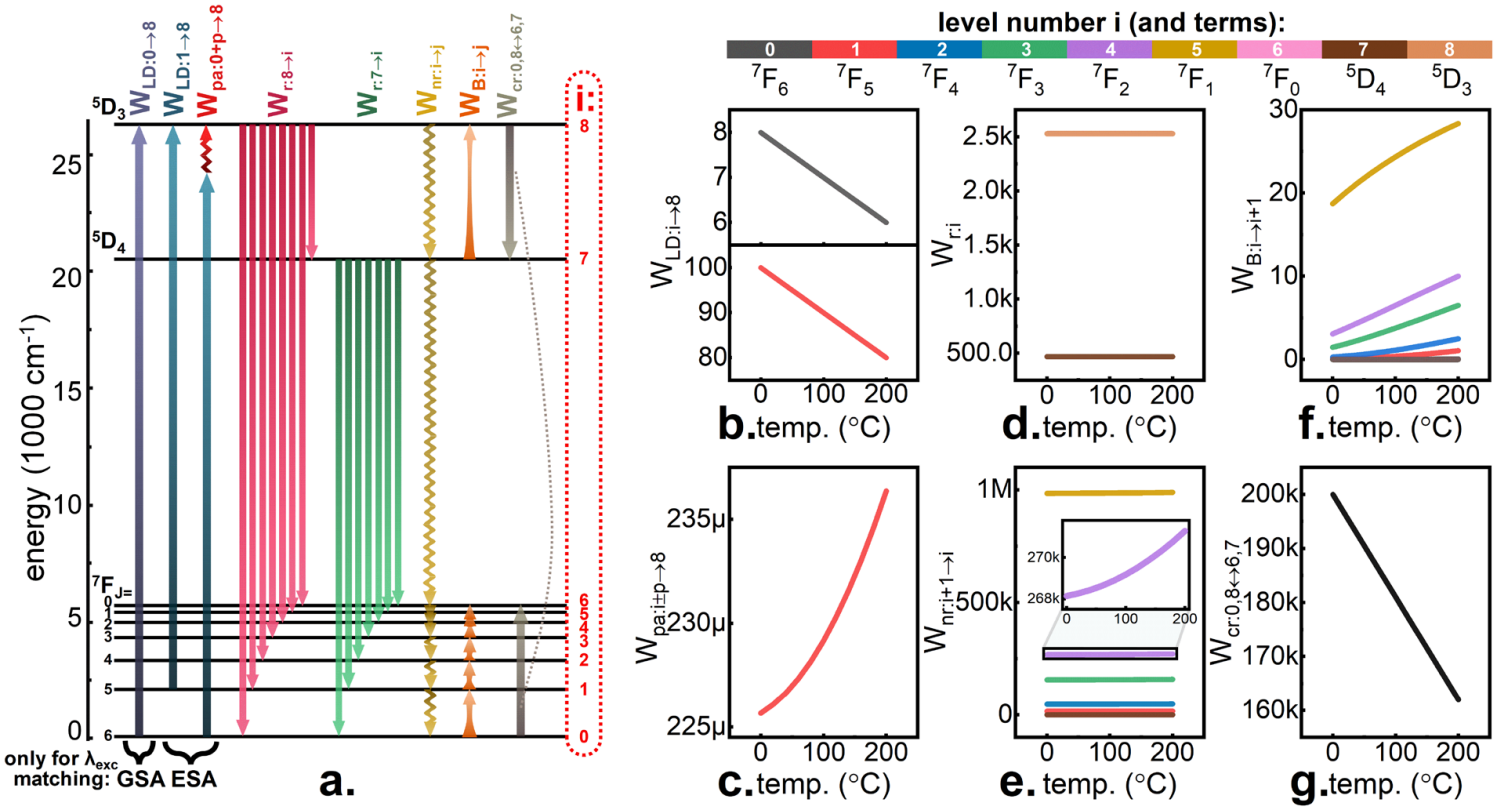
Energy level diagram of Tb^3+^ ion with all the investigated transitions and respective probabilities W marked—(**a**). Thermal evolution of following transition probabilities estimated for intermediate Tb^3+^ concentration: laser induced W_LD_—(**b**), phonon assisted W_pa_—(**c**), radiative W_r_—(**d**), nonradiative W_nr_—(**e**), Boltzmann distribution activated W_B_—(**f**), cross relaxation W_cr_—(**g**).

Processes whose probabilities *W*_*LD*_, *W*_*pa*_, *W*_*r*_ and *W*_*cr*_ depending on temperature are described by Eqs. 3, 4, 5 and 7, respectively, are schematically marked in Fig. 4a. The change of temperature may additionally affect the relative population of the energy states according to the Boltzmann distribution. The greater the gap between levels *∆E*_*ij*_, the less is the probability *W*_*B*_ of such transition:

8 $$W_{B:i \to j} = Ae^{{\frac{{ - \Delta E_{ij} }}{kT}}}$$

where for i = 0, …, 7: j = i + 1. Constant A does not depend on the concentration of dopant ions^6^ and its value should be around 50.

High temperature obviously facilitates the nonradiative depopulation of the energy states because the probability of the non-radiative transitions increases. The difference in energy *∆E*_*ij*_ between levels and maximum energy of matrix phonons *ω*_*max*_ that allow such transitions play an important role. The smaller the energy gap, the higher the probability *W*_*nr*_ of nonradiative transition. The dependence of *W*_*nr*_ on temperature is described by the energy gap rule:

9 $$W_{nr:i \to j} (T) = W_{nr:i \to j}^{T} = D_{1} e^{{ - D_{2} \Delta E_{ji} }} \left[ {1 - e^{{\frac{{ - \omega_{\max } }}{kT}}} } \right]^{{\frac{{ - \Delta E_{ji} }}{{\omega_{\max } }}}}$$

where for i = 1,…,8: j = i − 1.

To visualize how the values of individual probabilities depend on temperature, the thermal evolution of all the discussed *W* for examplamatory parameter values is presented in Fig. 4b–g. It should be emphasized that values of *W* do not correspond to the actual frequency of occurrence of a given kind of transition. These also depend on the occupation of the levels from which the transition takes place. *W*_*LD*_ (Fig. 4b) is shown for two laser lines: matched to GSA (377 nm) and matched to ESA (413 nm). As explained above, based on Judd–Ofelt parameters, in this case *W*_*LD:1*→*8*_ has higher values than *W*_*LD:0*→*8*_. However, it was assumed that the linear decrease of these probabilities similar because the electron–phonon coupling is equally dependent on temperature in case of both excitation lines. If a 413 nm excitation wavelength matched to ESA is used, another possible process has been described. The probability of a phonon assisted absorption *W*_*pa*_ increases with temperature in accordance with Eq. 4 as shown in Fig. 4c. Although the determined values are very small, they are rational because there are several orders of magnitude smaller than *W*_*LD*_ values. In addition, the frequency of occurrence of such transitions is still high, because it depends on population of the ground level which is always significant. The probabilities of radiative transitions *W*_*r*_ (Fig. 4d) are determined on the basis of the inverse of radiative lifetimes for levels ^5^D_3_ and ^5^D_4_. Because the lifetime of ^5^D_4_ is longer, the corresponding probability *W*_*r:7*_ is lower than the *W*_*r:8*_ that describe the emissions from ^5^D_3_ level. The values of these probabilities for subsequent transitions *W*_*r:i*→*j*_ are additionally scaled by branching ratios *β*_*i*→*j*_. As mentioned above, *W*_*r:i*→*j*_ are independent of temperature. Figure 4e shows the thermal dependence of the probabilities of nonradiative transitions *W*_*nr*_ between different levels. A strong dependence on the energy gap between levels is visible. The largest *W*_*nr*_ is achieved for the transition from ^7^F_0_ to ^7^F_1_. The smallest *W*_*nr*_ is for the transition between ^5^D_4_ and ^7^F_0_levels, because the gap in this case is the largest. The increase as a function of temperature is also visible for all *W*_*nr*_. A similar effect of dependence on the energy gap is observed for *W*_*B*_ (Fig. 4f). In this case, however, the increase as a function of temperature is more significant. Although the *W*_*B*_ values are relatively small, it is worth noting that these transitions occur from lower to higher levels, and thus—the first of them occurs from the ground level to the ^7^F_5_ level, which significantly increases its incidence and also affects the frequency of transitions between the above levels in a ladder-like process. The probability of transitions related to CR *W*_*cr*_ decreases linearly with temperature (Fig. 4g). The values of *W*_*cr*_ are very high, but in the case of such intraionic process, the frequency of this phenomenon depends on the population of both ground level and the ^5^D_3_ level, which is much lower, due to the rapid relaxation of the laser excited carriers via radiative and nonradiative path.

After analyzing all possible transitions between the levels marked with numbers from 0 for the ground level to 8 for ^5^D_3_ level, it was possible to create the following system of rate equations (Eqs. 10–18):

10 $$\frac{{dn_{0} }}{dt} = - W_{LD:0 \to 8}^{excGSA} n_{0} - W_{pa:0 + p \to 8}^{excESA} n_{0} + W_{r:7 \to 0} n_{7} + W_{r:8 \to 0} n_{8} + W_{nr:1 \to 0} n_{1} - W_{B:0 \to 1} n_{0} - W_{cr:0,8 \leftrightarrow 6,7} n_{0} n_{8}$$

11 $$\frac{{dn_{1} }}{dt} = - W_{LD:1 \to 8}^{excESA} n_{1} - W_{pa:1 - p \to 8}^{excGSA} n_{1} + W_{r:7 \to 1} n_{7} + W_{r:8 \to 1} n_{8} + W_{nr:2 \to 1} n_{2} - W_{nr:1 \to 0} n_{1} + W_{B:0 \to 1} n_{0} - W_{B:1 \to 2} n_{1}$$

12 $$\frac{{dn_{2} }}{dt} = W_{r:7 \to 2} n_{7} + W_{r:8 \to 2} n_{8} + W_{nr:3 \to 2} n_{3} - W_{nr:2 \to 1} n_{2} + W_{B:1 \to 2} n_{1} - W_{B:2 \to 3} n_{2}$$

13 $$\frac{{dn_{3} }}{dt} = W_{r:7 \to 3} n_{7} + W_{r:8 \to 3} n_{8} + W_{nr:4 \to 3} n_{4} - W_{nr:3 \to 2} n_{3} + W_{B:2 \to 3} n_{2} - W_{B:3 \to 4} n_{3}$$

14 $$\frac{{dn_{4} }}{dt} = W_{r:7 \to 4} n_{7} + W_{r:8 \to 4} n_{8} + W_{nr:5 \to 4} n_{5} - W_{nr:4 \to 3} n_{4} + W_{B:3 \to 4} n_{3} - W_{B:4 \to 5} n_{4}$$

15 $$\frac{{dn_{5} }}{dt} = W_{r:7 \to 5} n_{7} + W_{r:8 \to 5} n_{8} + W_{nr:6 \to 5} n_{6} - W_{nr:5 \to 4} n_{5} + W_{B:4 \to 5} n_{4} - W_{B:5 \to 6} n_{5}$$

16 $$\frac{{dn_{6} }}{dt} = W_{r:7 \to 6} n_{7} + W_{r:8 \to 6} n_{8} + W_{nr:7 \to 6} n_{7} - W_{nr:6 \to 5} n_{6} + W_{B:5 \to 6} n_{5} - W_{B:6 \to 7} n_{6} + W_{cr:0,8 \leftrightarrow 6,7} n_{0} n_{8}$$

17 $$\frac{{dn_{7} }}{dt} = - W_{r:7 \to \sum j} n_{7} + W_{r:8 \to 7} n_{8} + W_{nr:8 \to 7} n_{8} - W_{nr:7 \to 6} n_{7} + W_{B:6 \to 7} n_{6} - W_{B:7 \to 8} n_{7} + W_{cr:0,8 \leftrightarrow 6,7} n_{0} n_{8}$$

18 $$\frac{{dn_{8} }}{dt} = W_{LD:0 \to 8}^{excGSA} n_{0} + W_{LD:1 \to 8}^{excESA} n_{1} + W_{pa:0 + p \to 8}^{excESA} n_{0} + W_{pa:1 - p \to 8}^{excGSA} n_{1} - W_{r:8 \to \sum j} n_{8} - W_{nr:8 \to 7} n_{8} + W_{B:7 \to 8} n_{7} - W_{cr:0,8 \leftrightarrow 6,7} n_{0} n_{8}$$

These equations clearly show that the additional *W*_*cr*_ element can have a significant effect both on the intensity of the emission during stimulation adapted to ESA and on the variability of intensity as a function of temperature, because *W*_*cr*_ = *f*(*T*). Each CR act increases the population of the ^5^D_4_ level, which affects the intensity of emissions from this level. In addition, the population of the ^7^F_0_ level is increased, followed by very rapid non-radiative relaxation to the levels below, including the ^7^F_5_ level. Therefore, the probability of ESA process increases with the enhancement in probability of CR.

**Figure 5:**
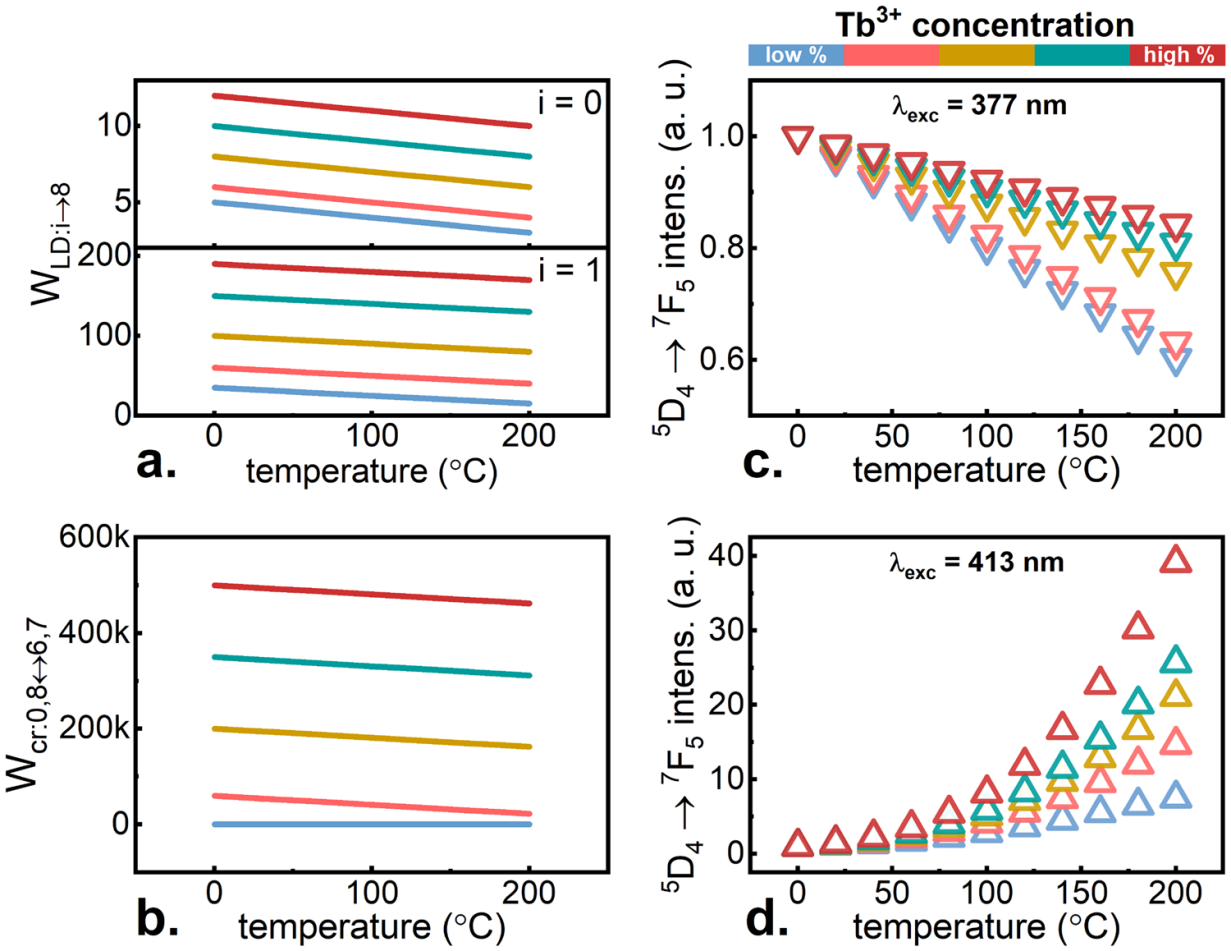
Thermal evolution of laser induced *W*_*LD*_ transition—(**a**) and cross relaxation induced transition—(**b**) probabilities estimated for KLa_1−x_Tb_x_P_4_O_12_ with different Tb^3+^ concentrations. Modeling results of ^5^D_4_ → ^7^F_5_ emission band intensity upon λ_exc_ = 377 nm—(**c**) and λ_exc_ = 413 nm—(**d**) excitation.

To determine the compliance of the model with experimental data, solving of the system of equations of the state described above with thermally dependent probabilities of transitions between levels *W* = *f*(*T*) was carried out. In this case, the initial conditions for temperature T = 0 °C were changed depending on the concentration of Tb^3+^ ions. First, it was assumed that as the content of Tb^3+^ ions in the matrix increases, the absorption cross section of the phosphor changes. Therefore, with a constant laser power, the probability of *W*_*LD:i*→*j*_, which is also a function of temperature, elevates with increasing Tb^3+^ concentration. Such changes in *W*_*LD:i*→*8*_ for both ESA (i = 0) and GSA (i = 1) excitation lines are shown in Fig. 5a. At the same time, as the concentration of Tb^3+^ increases, the *W*_*cr*_ also increases. It is responsible for changes in the overall luminescence intensity of nanocrystals and, surprisingly, it does not have a large impact on the shape of the obtained thermal curves of the intensity changes of individual bands. Assuming values and linear variations in the range corresponding to *W*_*cr*_ determined on the basis of experimental data (Eq. 6, Supplementary Fig. S3), some possible courses of the *W*_*cr*_ changes as a function of Tb^3+^ concentration are presented in Fig. 5b. Based on the data presented in Fig. 5a,b as well as the thermal evolution of other parameters *W*, which were considered independent of concentration, calculations were carried out. As a result, the ^5^D_4_ population variability as a function of temperature and concentration was obtained, which is linearly related to the observed ^5^D_4_ → ^7^F_5_ emission intensity that can be monitored in an experimental system around 543 nm. Data obtained using the 377 nm and 413 nm excitation lines are shown in Fig. 5c,d, respectively. In the case of GSA-matching excitation, a slight concentration relationship was noted in Fig. 5c. This is not in a great accordance with the experimental data, however, their normalization was not entirely rational, because each of the selected values was subject to erroneous measurement. The decrease in intensity over a temperature change from 0 to 200 °C, ranges from 40 to 20% of the initial value, which agrees with the experiment. For ESA-matched excitation, large changes in the shape of the curves were noted. For the modeled low Tb^3+^ concentration, assuming no CR and the smallest absorption cross-section, changes in intensity are less than tenfold. However, for a situation corresponding to a higher concentration, the increase in intensity over the investigated temperature range is greater. Both from the experiment and from the model it is clear that the highest Tb^3+^ concentration that can be achieved in nanocrystals with the total formula KTbP_4_O_12_ is optimal, because the sensitivity of the SBR nanothermometer based on this phosphor is very high and comparable with the previously described excitation wavelength selection case^6^, and thanks to the increase in the ratio signal-to-noise ratio and increasing the brightness of emissions, this case is a promising candidate for real applications.

Despite many advantages of SBR approach in luminescent thermometry its drawbacks should be mentioned here. The dispersive dependence of the medium's transmittance may affect the excitation intensity and hence the accuracy of temperature readout. However very similar artifact occurs in the case of the “regular” ratiometric luminescent thermometers, where the shape of one or both of bands may be partially absorbed, which can lead to an incorrect temperature determination. Therefore our further studies will be devoted to the elimination of this error by the implementation of the primary SBR luminescent thermometer. Furthermore, the intensity of the excitation emission using ESA-matched excitation wavelength is usually very low. A phosphor with very high emission brightness is therefore required to avoid this obstacle. Fortunately, this ESA-related effect is rather strong for KLa_1−x_Tb_x_P_4_O_12_ nanocrystals.

## Conclusions

This work describes extended research on luminescent nanothermometers in SBR approach concerning the enhancement of the temperature assessment sensitivity by the inter-ionic processes. The KLa_1−x_Tb_x_P_4_O_12_ nanocrystals were investigated, and the luminescence dynamics of ^5^D_4_ and ^5^D_3_ emitting states were presented and widely discussed, allowing the thermal dependence of probabilities of various processes on temperature to be estimated. The dependence of other processes on temperature was also proposed, basing on literature knowledge and a thorough analysis thorough analysis of experimental results. Thanks to this, the influence of Tb^3+^ concentration on the results obtained by KLa_1−x_Tb_x_P_4_O_12_ nanocrystals in the context of sensitivity to changes in ambient temperature has been discussed. The influence of the cross-relaxation process was analyzed, the main effect of which was a significant improvement in ^5^D_4_ emission brightness and the shift of the high relative sensitivities toward higher temperatures in respect to the direct ^5^D_4_ excitation.

In the SBR approach to luminescence thermometry, using only Tb^3+^ optically active ions in the simple nanocrystalline matrix, an innovative selection of wavelengths adapted to transitions ^7^F_6_ → ^5^D_3_ and ^7^F_5_ → ^5^D_3_ has been used. Thanks to this, it was possible to observe the intensity of the strong and spectrally distant ^5^D_4_ → ^7^F_5_ emission band, whose characteristics as a function of temperature turned out to be of an opposite nature for such excitation wavelengths. This band dominates the emission spectrum, which has contributed to improving luminescence brightness with non-resonant ESA-matched excitation, which is usually of very low intensity for lanthanide ions. What is more, the emission intensity was the higher the greater the Tb^3+^ ion content in the matrix. That is why the concentration of 100% Tb^3+^, occurring for KTbP_4_O_12_, proved to be the most optimal. In addition, this coincided with the maximum S_r_ obtained for this phosphor. S_r_ as high as 2–3%/°C was achieved in the whole tested temperature range, which is a significant value comparing to other SBR luminescent nanothermometers, that tend to have more restricted usable thermal ranges. Moreover, the maximum sensitivity of 3.2%/°C was achieved for a very high temperature of 190 °C, which increases the application possibilities of this phosphor in areas such as microelectronics.

## Supplementary information

Supplementary information
