# Supplementary material for: Enhancement of the sensitivity of single band ratiometric luminescent nanothermometers based on Tb3+ ions through activation of the cross relaxation process
Source: Sci Rep. 2020 Jul 7;10:11190. doi: 10.1038/s41598-020-68145-5 (PMC7341850; doi:10.1038/s41598-020-68145-5)
Supplement: Supplementary file 1 — Supplementary information [file 41598_2020_68145_MOESM1_ESM.docx]

**Supporting Information**

**Enhancement of the sensitivity of single band ratiometric luminescent nanothermometers based on Tb^3+^ ions through activation of the cross relaxation process**

**Joanna Drabik^1,^*, Robert Kowalski^1^, Lukasz Marciniak^1,^***

^1^Institute of Low Temperature and Structure Research, Polish Academy of Sciences, Okólna 2, 50-422 Wroclaw, Poland

*corresponding authors: *l.marciniak@intibs.pl, j.drabik@intibs.pl*


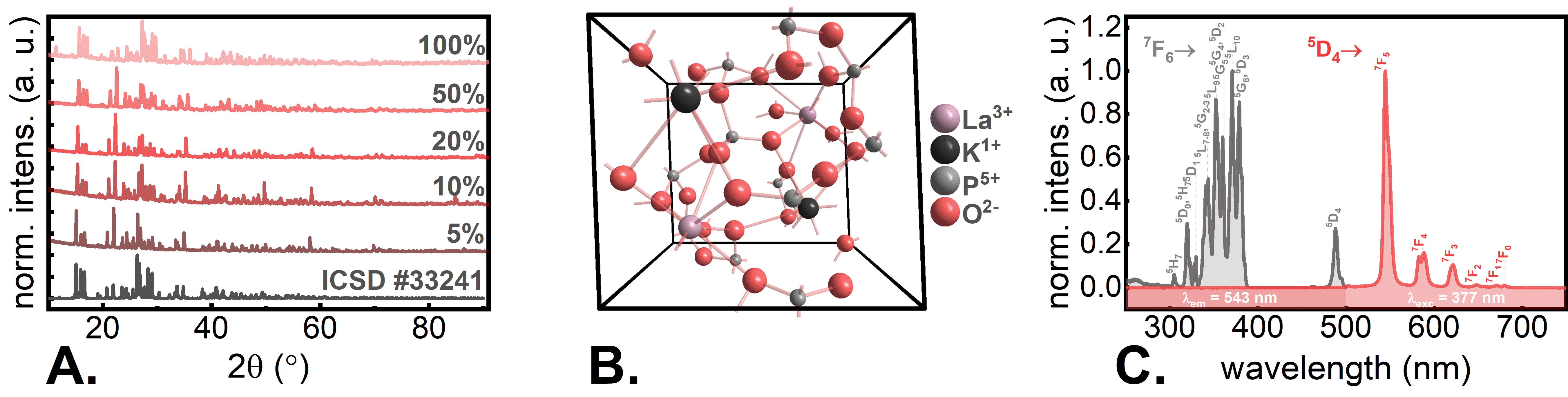


Figure S1. X-Ray Diffraction patterns of KLaP_4_O_12_:Tb^3+^ nanocrystals with different concentration of Tb^3+^ ions - (A). Unit cell of KLaP_4_O_12_ structure - (B); Representative room temperature excitation (measured using optical filter short-pass 500 nm) and emission spectra (measured using optical filter long-pass 500 nm) of KTbP_4_O_12_ – (C).


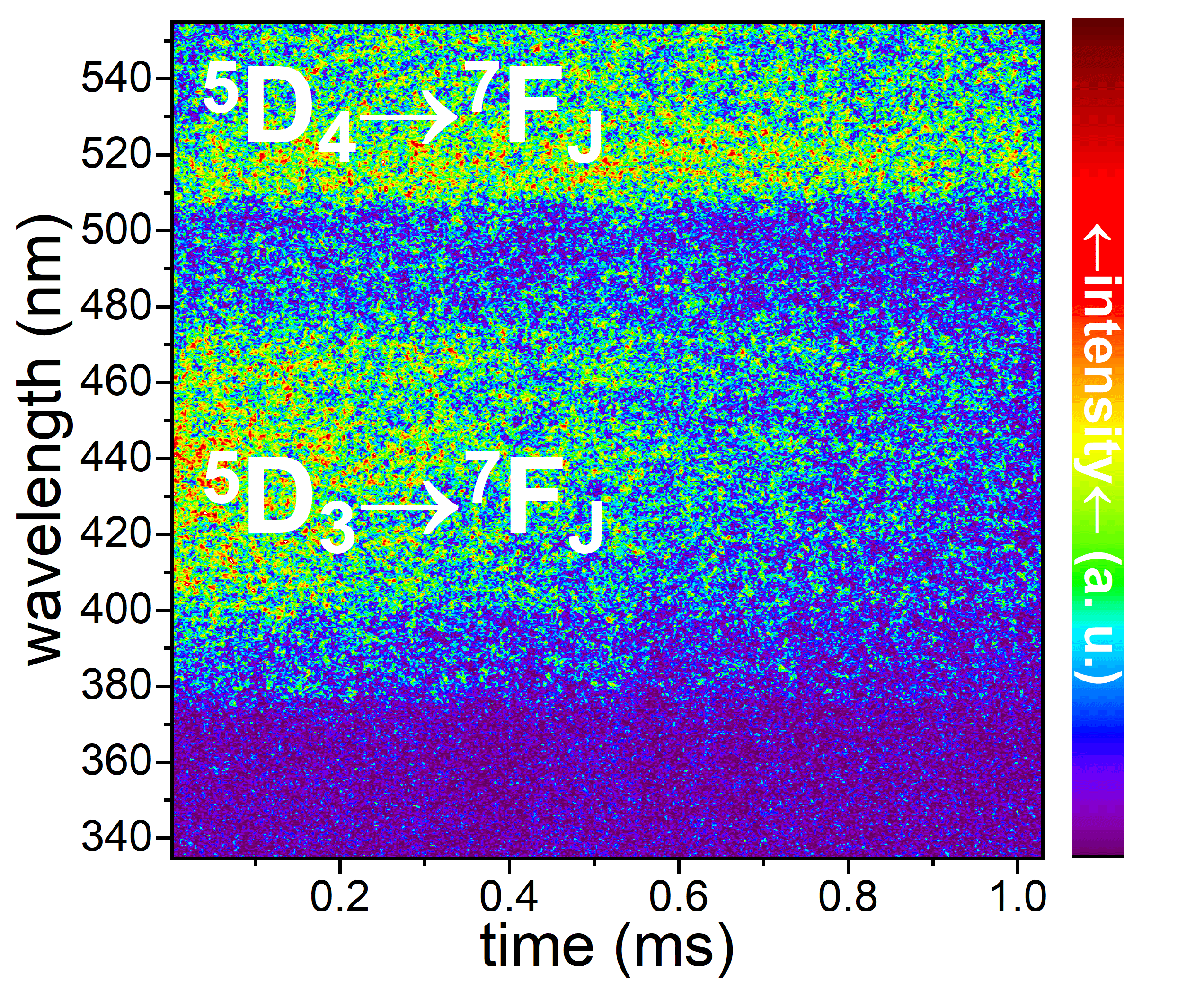


Figure S2. Representative room temperature time evolution of the emission intensity in a broad wavelength range for KLa_0.98_Tb_0.02_P_4_O_12_.

Table S1. Squared reduced-matrix elements $\left|\left\langle4f^{8}\alpha\left[SL\right]J\left\|U^{\left(t\right)}\right\|4f^{8}\alpha'\left[S'L'\right]J'\right\rangle\right|^{2}$ for intermanifold J→J’ transitions of Tb^3+^ ions. Table from ref. [15].

| **Transition** | **t = 2** | **t = 4** | **t = 6** |
| --- | --- | --- | --- |
| **^5^D_4_ → ^7^F_0_** | 0 | 0.0022 | 0 |
| **→ ^7^F_1_** | 0 | 0.0014 | 0 |
| **→ ^7^F_2_** | 0.0016 | 0.0004 | 8∙10^-5^ |
| **→ ^7^F_3_** | 0.0137 | 0.0010 | 0.0007 |
| **→ ^7^F_4_** | 0.0003 | 0.0019 | 0.0015 |
| **→ ^7^F_5_** | 0.0139 | 0.0010 | 0.0026 |
| **→ ^7^F_6_** | 0.0007 | 0.0013 | 0.0011 |
| **^5^D_3_ → ^5^D_4_** | 0.0501 | 0.0274 | 0.0063 |
| **→ ^7^F_0_** | 0 | 0 | 0 |
| **→ ^7^F_1_** | 0.0017 | 0.0030 | 0 |
| **→ ^7^F_2_** | 0.0016 | 0.0030 | 0 |
| **→ ^7^F_3_** | 0.0005 | 0.0111 | 0.0002 |
| **→ ^7^F_4_** | 0.0073 | 0.0002 | 0.0003 |
| **→ ^7^F_5_** | 0.0008 | 0.0028 | 0.0016 |
| **→ ^7^F_6_** | 0 | 0.0006 | 0.0014 |


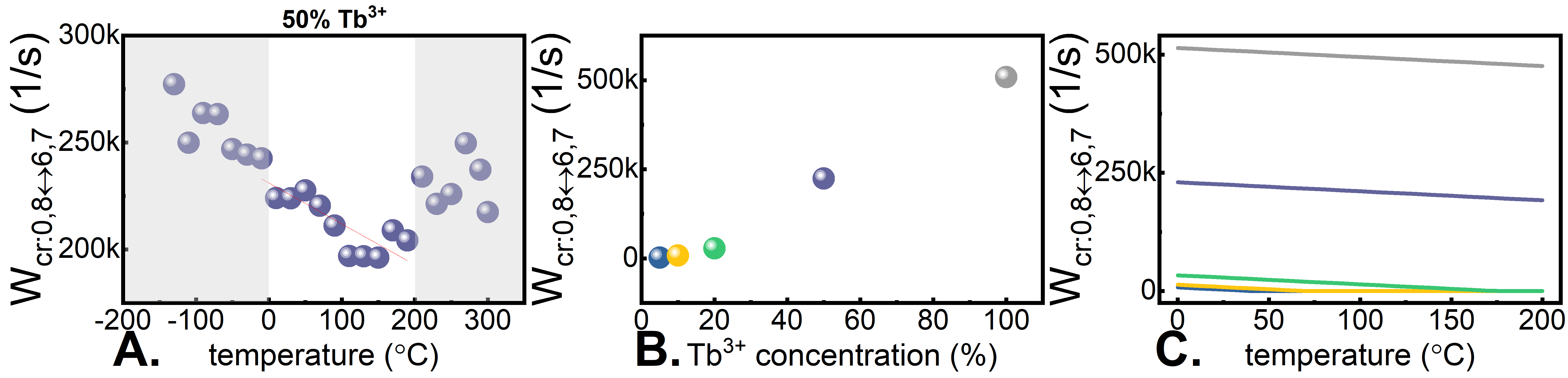


Figure S3. Calculated thermal evolution of the probability of the cross-relaxation process W_cr_ in a broad temperature range – (A). W_cr_ calculated at room temperature for all Tb^3+^ concentrations – (B). Extrapolated thermal dependence of W_cr_ for all Tb^3+^ concentrations – (C). Negative values were changed to W_cr_ = 0.
